# Development of InDel-Based Identification Methods for Wild and Domesticated Populations: A Case Study of *Larimichthys crocea*

**DOI:** 10.3390/ani16152339

**Published:** 2026-07-31

**Authors:** Zebin Zhou, Na Zhao, Meiqi Lv, Qianwen Min, Ruojing Li, Bi Huang, Jiayu Zhou, Xin Yi, Qiaomu Hu, Bo Zhang, Songlin Chen

**Affiliations:** 1 State Key Laboratory of Mariculture Biobreeding and Sustainable Goods, Yellow Sea Fisheries Research Institute, Chinese Academy of Fishery Sciences, Qingdao 266071, China; bangbinmen@163.com (Z.Z.); hqmu0806@163.com (Q.H.); 2 Southern Marine Science and Engineering Guangdong Laboratory (Zhanjiang), Zhanjiang 524059, China; penguinzn@163.com (N.Z.); lvmeiqi@zjblab.com (M.L.); minqianwen1996@163.com (Q.M.); liruojing@zjblab.com (R.L.); huangbi@zjblab.com (B.H.); 18475977935@163.com (J.Z.); yixin@zjblab.com (X.Y.); 3 Laboratory for Marine Fisheries Science and Food Production Processes, Qingdao Marine Science and Technology Center, Qingdao 266237, China

**Keywords:** whole-genome resequencing, InDel, genetic differentiation, classification method, large yellow croaker

## Abstract

The rapid expansion of aquaculture breeding has increased the need to identify wild genetic resources for germplasm management and sustainable breeding. However, distinguishing wild from domesticated populations remains challenging in species with recent domestication histories and low genetic differentiation. In this study, we used the large yellow croaker (*Larimichthys crocea*) as a case study species to develop specific insertion/deletion (InDels)-based identification methods for wild and domesticated populations based on whole-genome resequencing data. Four large-fragment InDels with significant allelic frequency differences between populations were selected as one marker panel. In addition, four coding-region InDels predicted to alter amino acid sequences were selected as another marker panel based on allele-frequency differences, coding-region annotation, and supporting SNP-based Fst or nucleotide-diversity signals. Principal component analysis (PCA) based on these selected InDel loci showed partial separation with some overlap between wild and domesticated samples. Therefore, multi-locus scoring models were established by assigning scores to wild-predominant alleles. Receiver operating characteristic (ROC) analysis showed strong discriminatory performance for both panels, with area under the ROC curve (AUC) values of 0.9608 and 0.9771 and classification agreement rates with known sample status of 90.0% and 91.7%, respectively. These results indicate that targeted InDel markers can provide a practical PCR-based approach for distinguishing wild and domesticated large yellow croaker populations with limited genetic differentiation. This workflow may provide a reference for marker development in other recently domesticated species, although further validation in independent populations and target species is needed.

## Simple Summary

Wild fish are important genetic resources for maintaining the long-term health of farmed populations. However, in species that have only been farmed for a relatively short time, wild and farmed individuals can be difficult to tell apart because their DNA differences are often limited. In this study, we used the large yellow croaker, an important marine fish in China, as a representative species to develop a DNA-based approach for identifying wild and domesticated populations. We selected several small insertion and deletion differences in DNA, known as InDels, that were more common in one population than in the other. Although plots based on these selected InDels still showed some overlap between the two groups, a multi-locus classification score based on their DNA patterns provided good discrimination between wild and domesticated individuals in the tested samples. This study provides a practical example of how a small set of targeted DNA differences can be used to identify populations with limited genetic differentiation. The general workflow may be adapted to other recently domesticated species after marker screening and validation in the target species.

## 1. Introduction

Distinguishing wild from domesticated populations is important for germplasm management, conservation of wild resources, and monitoring genetic exchange between farmed and natural populations. Molecular marker-based identification methods, including those based on single-nucleotide polymorphisms (SNPs) [1,2] and insertions/deletions (InDels) [3], provide objective tools for population assignment and support germplasm management and conservation genetics. However, in recently domesticated aquaculture species, genetic differentiation between wild and domesticated populations is often limited, making reliable identification challenging when only a small number of practical diagnostic markers are desired. Therefore, practical molecular markers are needed for population identification in such species [4].

The domestication of the large yellow croaker (*Larimichthys crocea*) began in the 1980s in southern China and has now spanned approximately 30 generations. The scale of large yellow croaker aquaculture has expanded significantly, making it one of the most important marine aquaculture fish species in China. However, in comparison to the common carp (*Cyprinus carpio*) [5] and Atlantic salmon (*Salmo salar*) [6], the large yellow croaker has undergone a relatively recent domestication process, and previous genetic studies have shown that differentiation between wild and domesticated populations remains limited [7]. Compared with species with long domestication histories, recently domesticated species often show smaller genetic differences between domesticated and wild populations. Previous studies on wild–domesticated population identification have mainly used SNP-based datasets or panels in aquaculture species, including blue catfish, white bass, and large yellow croaker [4,7,8]. These SNP-based approaches provide useful genome-wide information, but they often require sequencing, SNP arrays, or specialized genotyping platforms. In contrast, length-polymorphic InDel markers can be genotyped using conventional PCR and agarose gel electrophoresis when fragment-size differences are readily resolvable [9]. However, PCR-based InDel marker panels specifically designed for distinguishing wild and domesticated large yellow croaker populations with limited genetic differentiation remain insufficiently developed. This highlights the need for practical marker panels suitable for large yellow croaker populations with limited differentiation [10,11,12]. The large yellow croaker therefore provides a useful case study for developing identification methods for recently domesticated aquaculture species with limited differentiation. In such populations, different marker types may offer different practical advantages for population identification. Large-fragment InDels can facilitate rapid screening based on readily detectable length polymorphisms [13], whereas amino-acid-altering InDels in coding regions provide coding-region marker information. Therefore, these two marker categories were evaluated as alternative PCR-based approaches for population identification in this study.

To our knowledge, PCR-based InDel marker panels specifically developed and experimentally validated for distinguishing wild and domesticated large yellow croaker populations have not yet been reported. Here, this study aimed to develop PCR-based InDel marker panels for distinguishing wild and domesticated large yellow croaker populations by mining population-differentiated InDels from whole-genome resequencing data. By combining marker screening, experimental validation, and multi-locus scoring, this study may provide a practical workflow for developing targeted identification methods for recently domesticated species with limited genomic differentiation.

## 2. Materials and Methods

### 2.1. Sample Collection

This study collected 179 fin ray samples of wild large yellow croaker from various geographical locations for whole-genome resequencing, including the populations near Naozhou Island in Zhanjiang City, Guangdong Province, China (*n* = 63); the offshore areas of Liu’ao Town in Zhangpu City, Fujian Province, China (*n* = 60); and the coastal waters near the Pearl River Estuary in Guangdong Province, China (*n* = 56). Additionally, 216 fin ray samples of domesticated large yellow croaker were collected from different locations for whole-genome resequencing, sourced from Ningde City, Fujian Province, China (*n* = 60); Xiangshan Port, Zhejiang Province, China (*n* = 96); and Huizhou City, Guangdong Province, China (*n* = 60; in China, the production of domesticated large yellow croaker in the three provinces above accounts for 99.9% of the total output of this species [14]). These resequencing data were previously used for SNP-marker development [7] and were analyzed here for InDel-marker screening. This multi-region sampling design was intended to improve sample representation across major wild and domesticated sources. The wild or domesticated status of the samples was determined a priori based on documented sampling sources and collection records. To evaluate the potential influence of geographic background, genome-wide pairwise Fst values were calculated among the six sampling populations using quality-controlled SNPs and are provided in Supplementary Figure S1. This analysis was used only to assess population background and was not used for sample-label assignment or candidate InDel selection. In addition, a validation set comprising 120 wild and 120 domesticated samples was used to validate the PCR-based InDel marker panels. This set was independent of the resequencing marker-discovery samples and included the 60 wild and 60 domesticated validation samples used in the related SNP-marker study [7], together with additional samples. In this study, the sources of the large yellow croaker samples used for whole-genome resequencing and subsequent verification have been unambiguously identified. The large yellow croaker samples were collected using procedures approved by the Animal Experiment Review Committee of the Yellow Sea Fisheries Research Institute, Chinese Academy of Fishery Sciences.

### 2.2. Genomic DNA Extraction, Library Construction, and Sequencing

Genomic DNA was extracted from fin ray samples using the DNeasy Blood & Tissue Kit (QIAGEN, Hilden, Germany). Ribonuclease A was used to digest RNA in individual samples with RNA contamination, followed by genomic DNA extraction. Qualified genomic DNA samples were subjected to bead purification to remove impurities, including proteins, carbohydrates, and salts. The VAHTS Universal Plus DNA Library Prep Kit for Illumina V2 (Vazyme, Nanjing, China) was used for library construction. An appropriate amount of purified genomic DNA was used, and enzymatic fragmentation was performed in a suitable system to generate random genomic DNA fragments. The DNA fragments underwent end repair and 3′-end adenylation, followed by the attachment of adapters to the ends of these 3′-end adenylated fragments. The ligation products were subjected to bidirectional selection using magnetic beads to obtain genomic DNA fragments with an average insert size of 330–370 bp. The ligation products were PCR-amplified and purified to obtain the final libraries. Library quality was assessed using 2% agarose gel electrophoresis, which showed a main band at approximately 450–550 bp. The purified library was denatured into single-stranded DNA and cyclized through oligonucleotide sequences. Using single-stranded circular DNA (ssCir DNA) as a template, DNA nanospheres (DNB) were obtained through rolling-circle amplification. The DNB was loaded onto a microarray chip and sequenced using the MGISEQ-2000 (BGI Genomics, Shenzhen, China) in PE150 sequencing mode. The WGS data had an average sequencing depth of 13.27×, and the average Q30 score was greater than 95%.

### 2.3. Data Filtering and Alignment

Raw reads were filtered using SOAPnuke v2.1.7 [15]. The genome of the large yellow croaker (GCF_000972845.2) was downloaded from NCBI, and an index was constructed using BWA v0.7.15. The reads were aligned to the large yellow croaker reference genome using BWA-MEM [16]. On average, approximately 99.48% of the filtered reads were aligned to the reference genome.

### 2.4. Variant Site Calling, Conversion, and Annotation

Variant site detection was conducted using GATK v4.2.5.0 [17]. To construct an initial genome-wide InDel catalogue for variant characterization and annotation, vcftools was used for preliminary filtering with the following criteria: biallelic InDels, genotypes available in at least 80% of samples, genotype depth greater than 2, and minor allele frequency (MAF) ≥ 0.001. This permissive threshold was used only for initial variant cataloguing. A more stringent quality control specific for population differentiation analysis was applied subsequently (see Section 2.5). To perform variant annotation, the genome annotation file of the large yellow croaker was downloaded from the NCBI database (https://www.ncbi.nlm.nih.gov/datasets/genome/GCF_000972845.2/, accessed on 15 January 2026) [18]. ANNOVAR v2019Oct24 was used to annotate InDels, generating an initial dataset [19]. R v4.3.0 was used to visualize the InDel dataset.

### 2.5. Screening of Population-Differentiated Loci

The InDel dataset underwent quality control using PLINK v2.0 software with the parameters geno 0 and maf 0.05 [20]. PLINK v2.0 was used to calculate allele frequencies for wild and domesticated large yellow croaker populations. Allele frequency differences between wild and domesticated populations were tested using chi-square tests based on allele counts. To account for multiple testing, *p* values were adjusted using the Benjamini–Hochberg false discovery rate (FDR) method, and loci with both raw *p* < 0.001 and FDR-adjusted *p* < 0.05 were retained as population-differentiated candidate loci. For descriptive and analytical purposes, the identified InDels were categorized based on length: variants of 1–40 bp were classified as small InDels, whereas variants larger than 40 bp were classified as large-fragment InDels. This length threshold was used as a practical screening criterion because fragment-size differences above this range are more readily distinguished by conventional PCR and agarose gel electrophoresis under the validation conditions used in this study. The vcftools v 0.1.16 software was used to analyze the fixation index (Fst) and genetic polymorphism ratio (θπ) between the wild and domesticated large yellow croaker populations [21]. Fst measures population differentiation based on allele frequencies, while θπ reflects nucleotide diversity within a population. These metrics are widely used to detect genomic regions under selection. The top 2% Fst values and 5% *p*-values were used to identify selective signal regions in wild and domesticated large yellow croakers [22]. Genome-wide SNP-based Fst and nucleotide diversity analyses were used to provide supporting information for differentiated genomic regions. In this study, SNP-based Fst and nucleotide diversity signals were used as supporting prioritization evidence for coding-region InDels. Large-fragment InDels were selected mainly according to allele-frequency differences and readily resolvable fragment-length polymorphisms, whereas amino-acid-altering InDels were prioritized by combining allele-frequency differences, coding-region annotation, predicted amino acid alteration, and supporting SNP-based Fst or nucleotide diversity signals.

### 2.6. Principal Component Analysis Based on Differential Loci

Principal component analysis was performed using PLINK v2.0 based on the selected InDel marker panels. The large-fragment InDel panel and the amino-acid-altering InDel panel each included four loci, and the analyzed loci had no missing genotypes after filtering. PCA was conducted using the default PLINK allele-dosage coding and genotype standardization. The purpose of this analysis was to visualize the discriminatory pattern of the selected InDel markers between wild and domesticated samples.

### 2.7. Verification of Population-Specific Loci

Based on the screening of differential loci between wild and domesticated large yellow croaker populations, agarose gel electrophoresis marker primers were designed for the differential loci. An additional set of 120 fin DNA samples, each from wild and domesticated large yellow croakers, was used as templates for PCR-based genotyping. The reaction procedure was as follows: 95 °C for 10 min, followed by 95 °C for 30 s, 59 °C for 40 s, and 72 °C for 50 s, with 35 cycles, and then 72 °C for 5 min. After the reaction, electrophoresis was performed on a 2.5% agarose gel at 100 V for 35 min, and the electrophoresis pattern was captured using a gel imager. Genotypes were assigned according to the expected fragment-size differences or allele-specific amplification bands on agarose gels. The optimized primers and PCR conditions produced clear and interpretable banding patterns for all tested validation samples. Genotypes were assigned according to the expected fragment-size differences or allele-specific amplification bands on agarose gels, and PCR-based genotype calls were compared with the corresponding WGS-derived genotypes. For large-fragment InDels, genotypes were determined by fragment-size differences, whereas for amino-acid-altering InDels with small length differences, Del- and In-specific reactions were interpreted according to the presence or absence of expected allele-specific bands.

### 2.8. Scoring Model and ROC Analysis

To establish a simple and interpretable PCR-based classification rule, alleles enriched in the wild population were assigned a score of 1, whereas the alternative allele state was assigned a score of 0. All loci were given equal weight to avoid overfitting caused by the small number of markers and to maintain practical applicability for routine PCR-based genotyping. The total score of all alleles tested in each sample was calculated, and the total scores for each sample were summarized and grouped according to whether they were wild or domesticated large yellow croakers. The receiver operating characteristic (ROC) analysis was used to determine the classification threshold and evaluate discriminatory performance. The discriminatory performance of the scoring model was evaluated using the area under the ROC curve (AUC), 95% confidence interval (CI), sensitivity, specificity, and classification consistency. The optimal classification threshold was determined using the Youden index, calculated as sensitivity + specificity − 1. The independent validation set was not used for marker discovery and was used only to evaluate the PCR-based scoring model, thereby reducing the risk of overfitting.

### 2.9. Statistical Analyses

The chi-square test was used to compare allele frequencies between groups. The ROC curve analysis was performed to evaluate the discriminatory power of the genetic scoring model, and the AUC was calculated. Statistical analyses and graph generation were performed using GraphPad Prism 8.0 software. A stringent *p*-value threshold of <0.001 was applied to define statistical significance for all tests, to minimize false positives in the multiple-testing context. For ROC analysis, 95% CIs of AUC values were estimated using the nonparametric method.

## 3. Results

### 3.1. InDels Mining Based on Whole-Genome Resequencing Data

**Figure 1:**
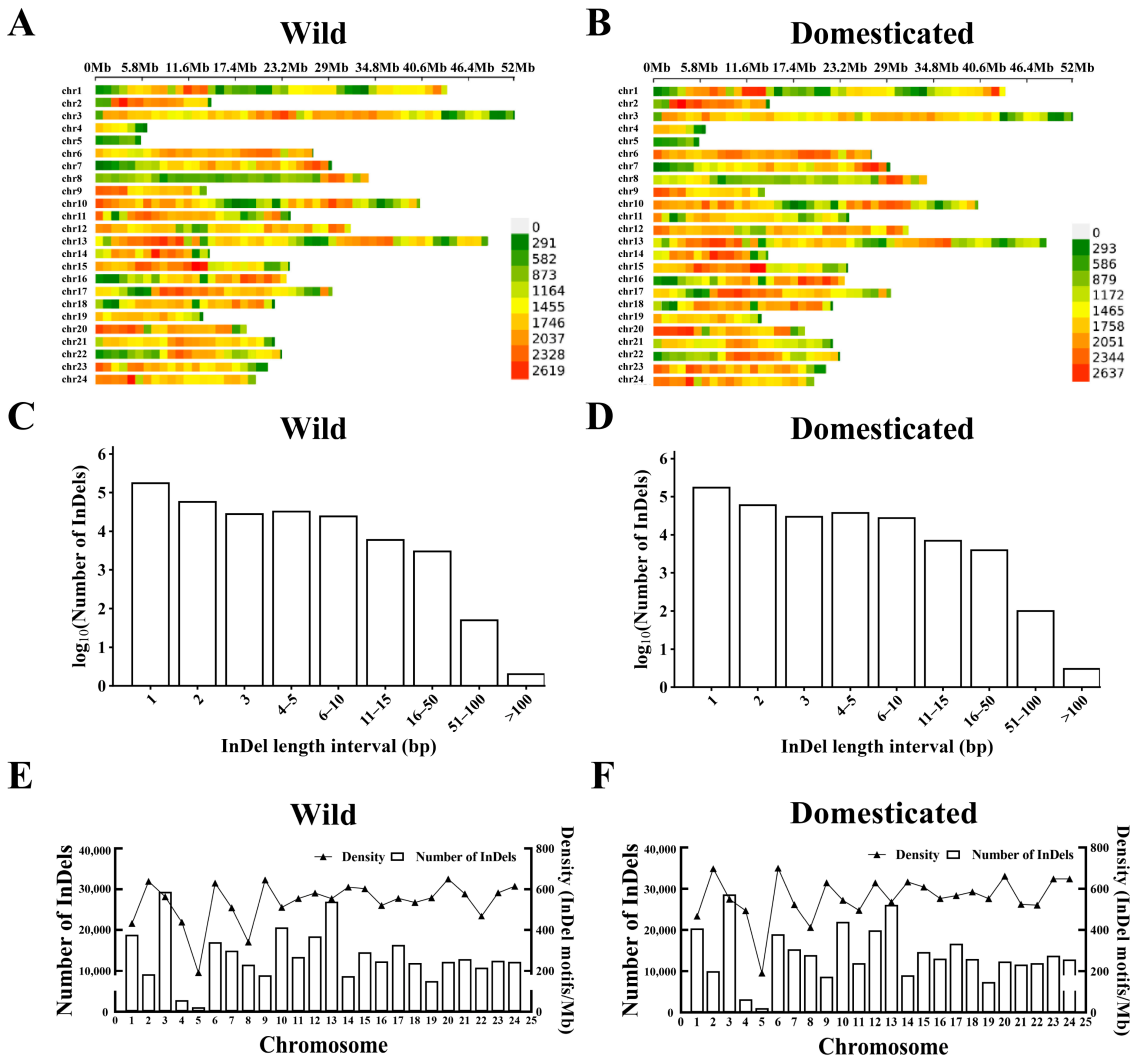
Chromosome distribution of InDels in the large yellow croaker. Density map of wild (**A**) and domesticated (**B**) large yellow croaker InDels on chromosomes. Warmer colors indicate higher InDel density; conversely, cooler colors indicate lesser InDel density. Relationship between the number and type of InDels in wild (**C**) and domesticated (**D**) large yellow croaker. Density histogram of wild (**E**) and domesticated (**F**) large yellow croaker InDels on chromosomes.

After read alignment and variant calling, 18,279,565 raw InDel candidates were obtained as the initial dataset. After population-specific quality filtering described in the Materials and Methods, 325,830 InDels in the wild population and 337,141 InDels in the domesticated population were retained for downstream descriptive analyses. The filtered InDel density plots showed the distribution of InDel markers on each chromosome for wild and domesticated large yellow croaker populations (Figure 1A,B). The density plots showed high- and low-density regions of InDels across chromosomes in both wild and domesticated populations, with relatively low-density regions observed on chromosome 5 in both groups. The sizes of InDels in the wild large yellow croaker population ranged from 1 to 146 bp, with fewer InDels at longer lengths. Among them, 89.74% of InDels were five bp or less, 9.32% were 6–15 bp, and 0.95% were more than 15 bp. Among all InDels, the most common type was mononucleotide variants, accounting for 53.64% (174,548). Additionally, dinucleotides (57,303) and trinucleotides (27,828) accounted for 17.61% and 8.55%, respectively (Figure 1C). The size range of InDels in the domesticated large yellow croaker population was also 1–146 bp, with most InDels concentrated in the short-length categories. Among them, 88.57% of InDels were five bp or less, 10.23% were 6–15 bp, and 1.20% were more than 15 bp. The most abundant type of InDels was mononucleotides, comprising 171,792 (50.96%). In the domesticated group, the dinucleotide (59,707) and trinucleotide (29,700) InDels accounted for 17.71% and 8.81%, respectively (Figure 1D). Wild large yellow croakers showed an average genome-wide InDel density of 536.31/Mb, ranging from 341.27 (chr8) to 650.49 (chr20) (Figure 1E). The domesticated population showed an average genome-wide InDel density of 556.99/Mb, ranging from 411.45/Mb on chromosome 8 to 700.39/Mb on chromosome 6 (Figure 1F).

**Figure 2:**
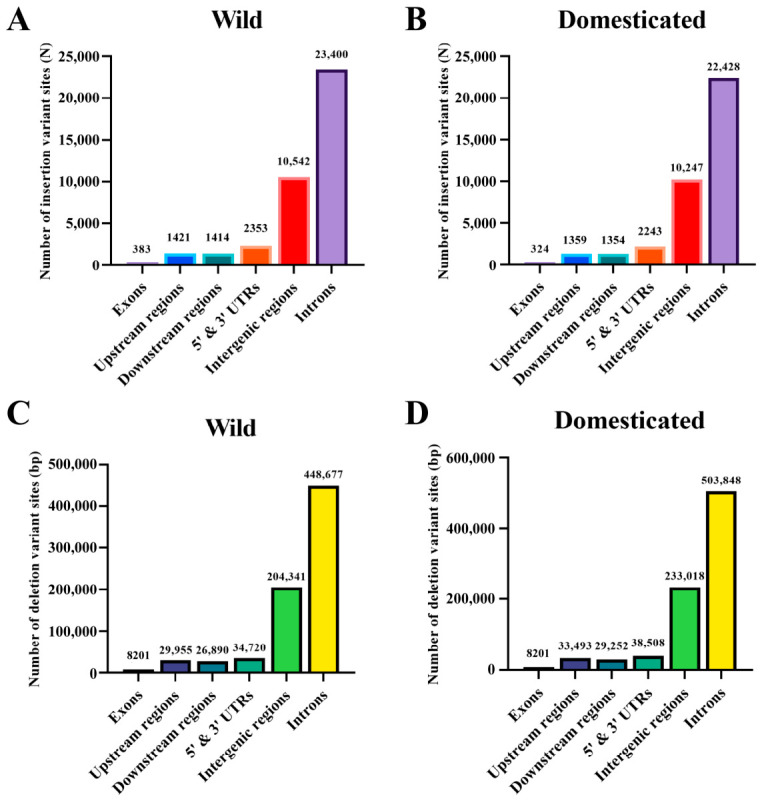
The relationship between the Location of InDels and Genes in the large yellow croaker. Classification and number of insertion mutation sites in wild (**A**) and domesticated (**B**) large yellow croaker. Classification of deletion mutation sites and number of deleted bases in wild (**C**) and domesticated (**D**) large yellow croaker. These annotation results were used to provide a descriptive overview of the genomic locations of InDels, rather than to infer functional-category enrichment.

Based on the filtered population-specific InDel datasets, insertion and deletion events were further annotated according to their genomic locations. In the wild large yellow croaker population, 39,513 insertion sites were obtained, of which 59.22% were in introns, 26.68% in intergenic regions, 6.00% in 5′UTR or 3′UTR, 3.60% in upstream regions of genes, 3.58% in downstream regions of genes, and 0.97% in exons (Figure 2A). Furthermore, we categorized the InDel set based on insertion and deletion sites. In the domesticated population, 37,955 insertion sites were obtained, of which 59.09% were in introns, 27.00% in intergenic regions, 5.91% in 5′UTR or 3′UTR, 3.58% in upstream regions of genes, 3.57% in downstream regions of genes, and 1.01% in exons (Figure 2B). Given that the deletion sites in the InDel dataset span multiple functional regions of the genome, statistical analysis was conducted on the actual positions of the deleted bases. In the wild population, a total of 752,784 bp of deleted bases were identified, with 59.60% located in introns, 27.14% in intergenic regions, 4.61% in 5′UTR or 3′UTR, 3.98% in upstream regions of genes, 3.57% in downstream regions of genes, and 1.09% in exons (Figure 2C). Similarly, in the domesticated population, a total of 846,320 bp of deleted bases were identified, with 59.53% located in introns, 27.53% in intergenic regions, 4.55% in 5′UTR or 3′UTR, 3.96% in upstream regions of genes, 3.46% in downstream regions of genes, and 0.97% in exons (Figure 2D).

### 3.2. Differential Loci Screening and Principal Component Analysis

A total of seven large-fragment InDels met the screening criteria of allele-frequency difference greater than 0.50 between wild and domesticated populations and fragment length greater than 40 bp. After primer design and PCR optimization, four loci produced stable, clear, and agarose-resolvable banding patterns and were retained for PCR validation. These loci exhibited significant divergence (*p* < 0.001) between wild and domesticated populations and had fragment-length differences of 49–54 bp, which were suitable for direct PCR-gel electrophoresis validation (Table 1).

For the amino-acid-altering InDel panel, 101 coding-region InDels with predicted amino acid alterations were identified with supporting SNP-based Fst or nucleotide-diversity signals. The 20 loci with the largest allele-frequency differences between wild and domesticated populations were selected for primer design and PCR optimization. Among them, four loci produced stable and interpretable PCR-gel electrophoresis patterns using allele-specific PCR and were retained as amino-acid-altering InDels (Table 2). Based on gene annotations, these loci were associated with genes involved in cytokine receptor signaling, p53-related kinase binding, ARF GTPase regulation, and lysosomal proteolysis. SNP-based Fst and nucleotide-diversity signals overlapping or near these loci are summarized in Table S1.

**Figure 3:**
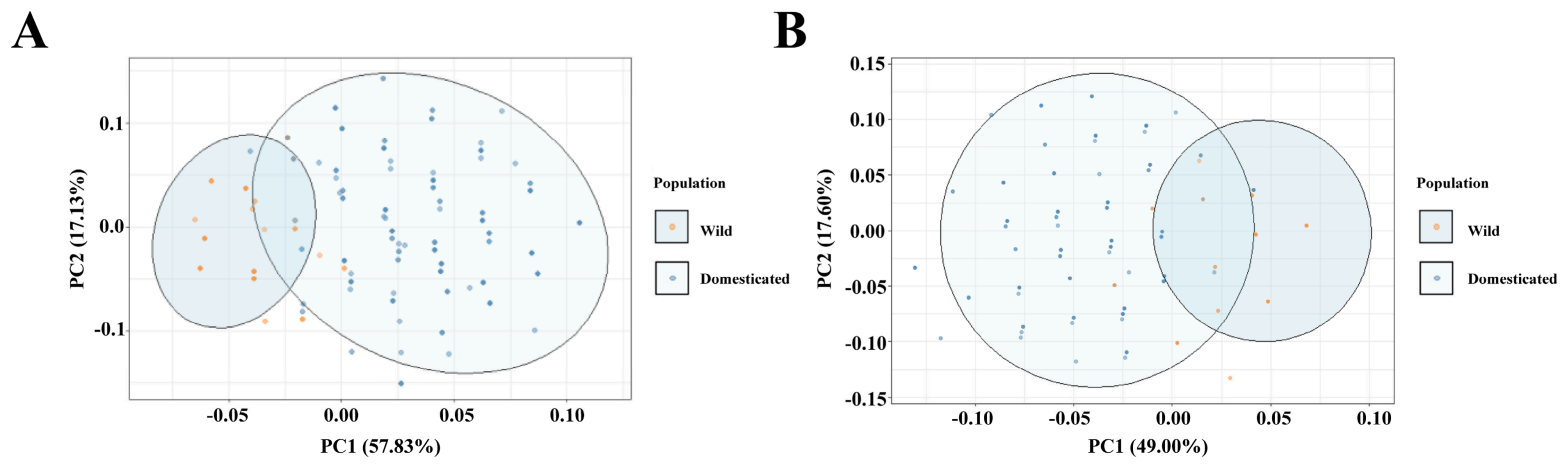
PCA visualization of wild and domesticated large yellow croaker based on selected InDel marker panels. (**A**) Large-fragment InDel panel. (**B**) Amino-acid-altering InDel panel. Both panels showed population-related grouping trends, but visual overlap remained between wild and domesticated samples.

For the large-fragment InDel panel, PCA showed a separation trend between wild and domesticated samples, with PC1 and PC2 explaining 57.83% and 17.13% of the variation, respectively; however, the two groups were not completely separated in the PCA plot (Figure 3A). UMAP analysis based on the same marker panel showed a broadly consistent grouping pattern, but visual overlap between groups was still observed (Figure S2A). For the amino-acid-altering InDel panel, PCA also showed partial separation between wild and domesticated samples, with PC1 and PC2 explaining 49.00% and 17.60% of the variation, respectively (Figure 3B), and UMAP showed a comparable grouping trend (Figure S2B). These results indicate that the selected InDel loci captured population-related differences, but visual ordination alone was insufficient for complete individual classification. Therefore, classification performance was further evaluated using the multi-locus scoring model and ROC analysis.

### 3.3. Verification of Differential InDels

**Figure 4:**
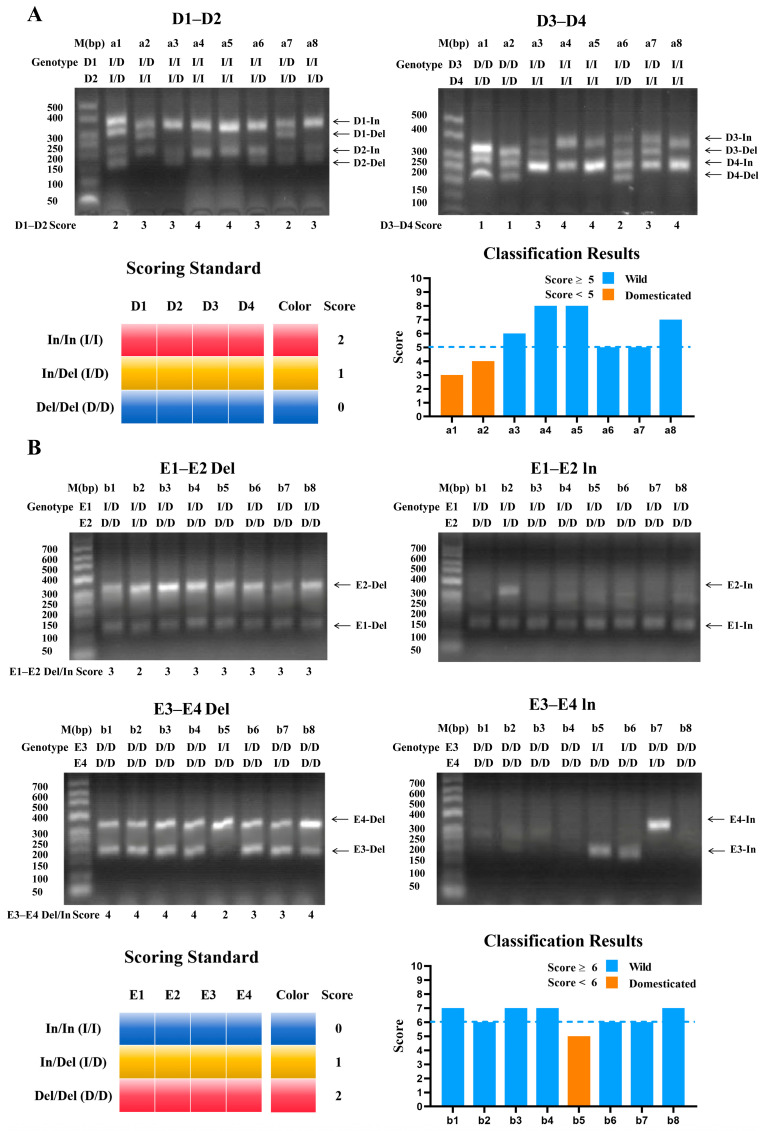
Validation of InDel differences between wild and domesticated large yellow croaker. The genotype-specific scoring values for the InDel are listed below the agarose gel electrophoresis pattern. Taking samples 1–8 as examples, agarose gel electrophoresis verification was conducted based on large-fragment InDels. (**A**) Agarose gel electrophoresis pattern D1–D2 shows the In and Del genotypes at D1 and D2 (when both In and Del bands are present in the same lane, the genotype of the sample is In/Del; when only In band is present, the genotype of the sample is In/In; when only Del band is present, the genotype of the sample is Del/Del). The agarose gel electrophoresis pattern D3–D4 shows the In and Del genotypes at D3 and D4. (**B**) Similarly, using samples 1–8 as examples, agarose gel electrophoresis verification was conducted for the amino-acid-altering InDels. Agarose gel electrophoresis pattern E1–E2 Del and E1–E2 In, respectively, show the In and Del genotypes at the first two InDels of the system. The agarose gel electrophoresis pattern E3–E4 Del and E3–E4 In shows the In and Del genotypes at the first two InDels of the system.

PCR validation of the large-fragment InDels produced clear fragment-size differences corresponding to In and Del alleles (Figure 4A, Tables S2 and S3). The genotype frequencies obtained from PCR-based validation were generally consistent with the resequencing-based allele-frequency patterns in the sampled populations (Table 3).

PCR validation of the amino-acid-altering InDels showed allele-specific amplification patterns for the In and Del alleles (Figure 4B). The validated genotype frequencies were consistent with the resequencing-based allele-frequency patterns in the sampled populations (Table 4). The PCR genotyping results matched the corresponding WGS genotypes for the tested loci.

### 3.4. Construction of the Screening Method and Verification of the Method’s Applicability

**Figure 5:**
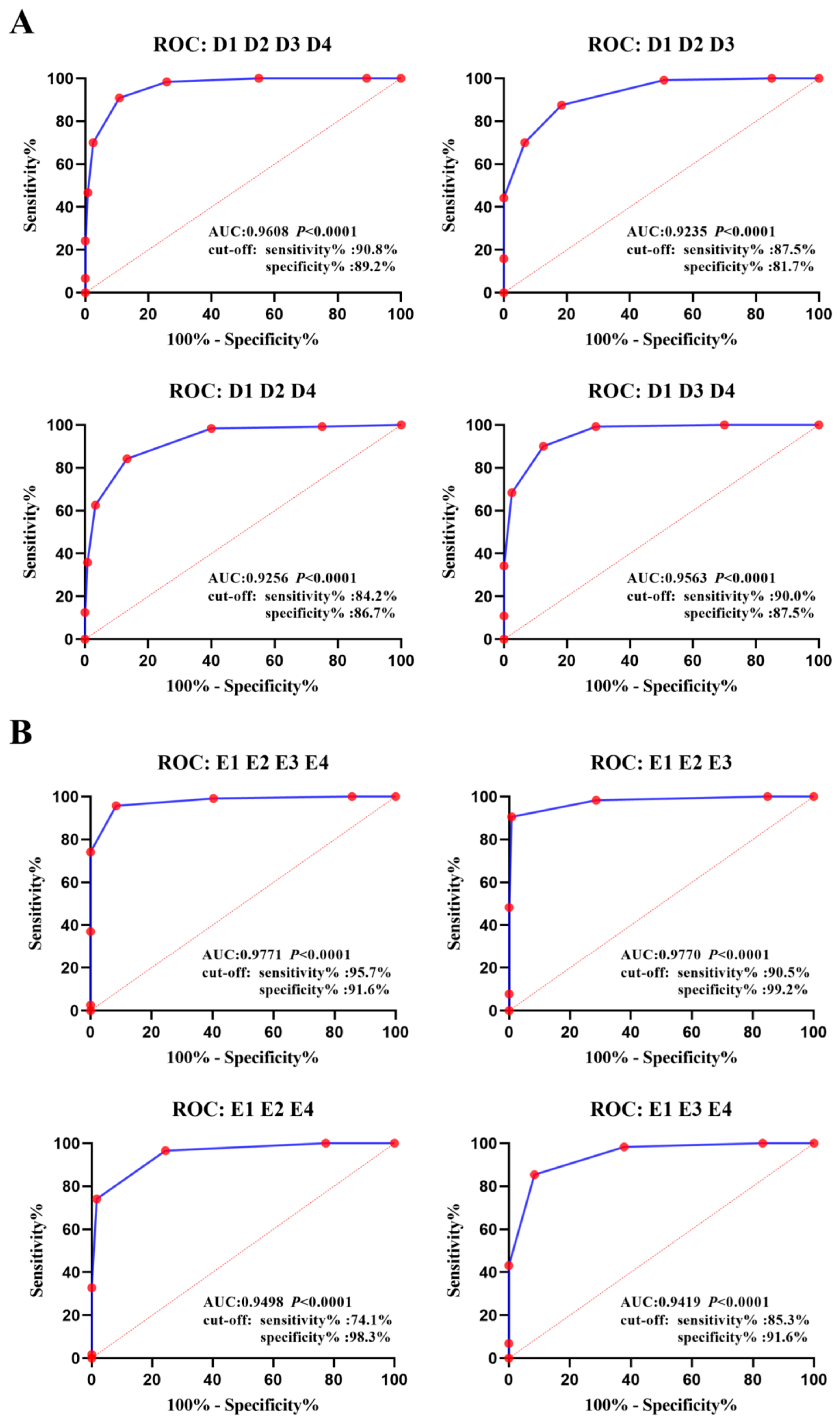
ROC evaluation of the selected InDel marker panels for distinguishing wild and domesticated large yellow croaker. (**A**) ROC curves for the large-fragment InDel panel and representative marker combinations. (**B**) ROC curves for the amino-acid-altering InDel panel and representative marker combinations. AUC, sensitivity, specificity, and optimal thresholds are shown for each model.

ROC analysis was performed using the PCR validation results of the large-fragment InDel panel. Different marker combinations were evaluated, and the four-locus panel produced the highest AUC among the tested combinations (Figure 5A). Based on the equal-weight scoring rule described above, the optimal threshold was determined by maximizing Youden’s index. The four-locus panel had an AUC of 0.9608 (95% CI: 0.9394–0.9822). At the threshold of 5, the sensitivity (i.e., the probability of correctly identifying a wild large yellow croaker with this typing method) and specificity (i.e., the likelihood of correctly excluding a domesticated large yellow croaker with this typing method) were 90.8% (95% CI: 84.33–94.80%) and 89.2% (95% CI: 82.34–93.56%), respectively. Samples with scores greater than 5 were classified as wild large yellow croaker, whereas those with scores of 5 or lower were classified as domesticated samples. The classification consistency between the scoring-based identification results and the known sample status was 90.0%.

Similarly, ROC analysis was performed using the PCR validation results of the amino-acid-altering InDel panel. Among the tested marker combinations, the four-locus panel produced the highest AUC (Figure 5B). Based on the same threshold-selection criterion, the four-locus panel had an AUC of 0.9771 (95% CI: 0.9609–0.9903). At the threshold of 6, the sensitivity and specificity were 95.7% (95% CI: 90.31–98.15%) and 91.6% (95% CI: 85.22–95.37%), respectively. Samples with scores greater than 6 were classified as wild large yellow croaker, whereas those with scores of 6 or lower were classified as domesticated samples. The classification consistency between the scoring-based identification results and the known sample status was 91.7%. Together, these results indicate that both four-locus panels showed strong and generally comparable discriminatory performance in the validation samples.

## 4. Discussion

This study used short-read whole-genome resequencing to identify genome-wide InDel variation in wild and domesticated large yellow croaker and to construct an InDel dataset for marker screening. The Illumina-based sequencing workflow provides high-throughput genome-wide data suitable for initial population-level marker screening [23].

The detected InDels showed uneven distribution patterns across chromosomes. Their chromosomal distribution may be associated with local genomic characteristics. For example, repeat-rich regions, including tandem repeats and transposable elements, may contribute to local InDel accumulation [24], whereas recombination-related repair processes may influence regions with lower InDel density [25]. In both wild and domesticated large yellow croaker populations, InDels were more frequently located in intronic and intergenic regions than in exonic regions, suggesting stronger purifying selection against potentially deleterious exonic variants [26].

Two marker panels were evaluated in this study: a large-fragment InDel panel and an amino-acid-altering InDel panel. Principal component analysis showed that both panels separated most wild and domesticated samples, although a small overlap remained between the two groups. The small overlap between the two populations in principal component analysis reflects incomplete discrimination power of the selected marker panels. Thus, the classification performance of each panel was further evaluated using the multi-locus scoring model and ROC analysis. The large-fragment InDel panel and the amino-acid-altering InDel panel provide two separate PCR-based marker options for distinguishing wild and domesticated large yellow croaker populations.

Precise identification of wild and domesticated populations is particularly important for recently domesticated species because it supports germplasm management and conservation. Traditional approaches, such as morphological characteristics [27], high-density SNP arrays [28] and mitochondrial haplotype analysis [29,30], may be limited by growth stage, platform requirements, maternal inheritance, or low resolution. In this study, two PCR-based InDel marker panels were developed using conventional PCR-gel electrophoresis and a multi-locus scoring strategy. These panels may improve population identification in large yellow croaker with limited genetic differentiation. Compared with methods relying on microsatellites [31] or mitochondrial markers [32], these panels provide a multi-locus PCR-based option for recently domesticated species such as large yellow croaker.

The two InDel marker panels developed in this study have different practical features and application scenarios. The large-fragment InDel panel is based on large InDels with marked allele-frequency differences and facilitates rapid screening because its alleles can be directly distinguished by fragment-length differences [33]. The amino-acid-altering InDel panel provides coding-region marker information. These amino-acid-altering InDels may represent candidate coding-region markers associated with population differentiation. Some loci were annotated to genes such as interleukin-31 receptor subunit alpha (IL-31RA) and TP53-regulated kinase-binding protein (TP53RKB). Their biological effects require further validation through phenotypic, expression, or trait-association analyses. Both InDel marker panels developed in this study are PCR-gel electrophoresis-based genotyping assays [34,35,36,37]. The large-fragment InDel panel uses genomic DNA as the template and is suitable for routine PCR-based screening because its alleles can be directly distinguished by fragment-length differences. The amino-acid-altering InDel panel provides coding-region marker information, but it requires separate Del- and In-specific reactions because of the small InDel sizes, leading to more PCR reactions and stricter amplification conditions [38]. Therefore, the two panels are not mandatory combined tests for every sample; instead, they provide flexible alternatives that can be selected according to template type and research purpose. This PCR-based application framework provides a practical alternative for situations where high-density SNP arrays or sequencing-based genotyping platforms are less accessible [39]. The candidate loci were identified from resequencing data and evaluated by PCR-based genotyping in an independent validation set, supporting their use for population discrimination in the sampled large yellow croaker populations. Because the panels were evaluated using a single validation set, the performance estimates should be interpreted cautiously, and the panels should be tested in additional independent populations. Future studies could further evaluate this marker-development framework in additional populations and other recently domesticated species with limited genetic differentiation.

## 5. Conclusions

This study characterized genome-wide InDel variation in resequencing data from wild and domesticated large yellow croaker and developed two small PCR-based InDel marker panels: a large-fragment InDel panel and an amino-acid-altering InDel panel. In the validation samples, the two panels showed good discriminatory performance, with AUC values of 0.9608 (95% CI: 0.9394–0.9822) and 0.9771 (95% CI: 0.9609–0.9903), and classification consistencies of 90.0% and 91.7%, respectively. These results suggest that selected InDel markers can provide a practical PCR-based approach for distinguishing wild and domesticated large yellow croaker populations in the tested populations. Future studies applying this marker-development framework to additional populations and other recently domesticated species will help further evaluate its broader applicability in populations with limited genetic differentiation.

## Figures and Tables

**Table 1:** Large-fragment InDels between wild and domesticated large yellow croaker.

InDel Number	InDel Position (Chromosome-Position)	Allele Type	Population with Higher Allele Frequency	The Absolute Value of the Allele Frequency Difference Between Populations	*p* Value	InDel (bp)
D1	21-17662246	Del	Domesticated	0.533845	3.894 × 10^−51^	52
D2	21-13979637	Del	Domesticated	0.5193839	2.216 × 10^−54^	54
D3	1-35741636	Del	Domesticated	0.532928	3.729 × 10^−47^	52
D4	3-21732774	Del	Domesticated	0.509694	8.869 × 10^−55^	49

**Table 2:** Amino-acid-altering InDels between wild and domesticated large yellow croaker.

ID	InDel Position	Allele Type	Population with Higher Allele Frequency	Absolute Value of Allele Frequency Difference	*p* Value	Site Mutation	InDel-Associated Gene
E1	11-5053756	In	Domesticated	0.556073	1.579 × 10^−54^	AG → A	Interleukin-31 receptor subunit alpha (IL-31RA)
E2	20-14004876	In	Domesticated	0.4082035	1.325 × 10^−39^	AT → A	TP53-regulated kinase binding protein (TP53RKB)
E3	3-28345321	In	Domesticated	0.366323	9.880 × 10^−27^	GT → G	ARF GTPase-activating protein 2 (GIT2)
E4	21-17327392	In	Domesticated	0.3320665	5.993 × 10^−27^	TCAAA → T	cathepsin H (ctsh)

**Table 3:** PCR-based genotype validation of large-fragment InDels.

InDel Number	InDel Position (Chromosome-Position)	Wild Samples, *n* (%)			Domesticated Samples, *n* (%)		
		In/In	In/Del	Del/Del	In/In	In/Del	Del/Del
D1	21-17662246	79/65.8%	34/28.3%	7/5.8%	12/10%	50/41.7%	58/48.3%
D2	21-13979637	56/46.7%	44/36.7%	20/16.7%	15/12.5%	49/40.8%	56/46.7%
D3	1-35741636	65/54.2%	48/40%	7/5.8%	10/8.3%	47/39.2%	63/52.5%
D4	3-21732774	99/82.5%	14/11.7%	7/5.8%	22/18.3%	52/43.3%	46/38.3%

**Table 4:** PCR-based genotype validation of amino-acid-altering InDels.

InDel Number	InDel Position (Chromosome-Position)	Wild Samples, n (%)			Domesticated Samples, n (%)		
		In/In	In/Del	Del/Del	In/In	In/Del	Del/Del
E1	11-5053756	7/5.9%	79/66.4%	33/27.7%	79/68.1%	35/30.2%	2/1.7%
E2	20-14004876	0/0%	6/5.0%	113/95.0%	13/11.2%	61/52.6%	42/36.2%
E3	3-28345321	1/0.8%	35/29.4%	83/69.7%	24/20.7%	72/62.1%	20/17.2%
E4	21-17327392	0/0%	24/20.2%	95/79.8%	0/0%	74/63.8%	42/36.2%

One wild and four domesticated validation samples were excluded from the amino-acid-altering InDel panel because of inconsistent amplification across loci.

## Data Availability

The raw data supporting the conclusions of this article will be made available by the authors on request.

## Supplementary Materials

The following supporting information can be downloaded at: https://www.mdpi.com/article/10.3390/ani16152339/s1, Table S1. SNP-based Fst and nucleotide diversity signals associated with the four selected amino-acid-altering InDels. Table S2. Amplification primers used to verify the different InDels between wild and domesticated large yellow croaker based on allele frequency and chi-square test (*p* < 0.001). Table S3. Amplification primers for differentiated InDels between wild and domesticated large yellow croaker based on verification and selective signal analysis. Figure S1. Genome-wide pairwise Fst among the six sampling populations of large yellow croaker used for marker discovery. Pairwise FST values were calculated using quality-controlled genome-wide SNPs. ZJKW, ZPW, and NZDW represent wild populations collected from the coastal waters near the Pearl River Estuary, the offshore areas of Liu’ao Town in Zhangpu City, and the waters near Naozhou Island, respectively. GDD, FJD, and ZJD represent domesticated populations from Guangdong, Fujian, and Zhejiang Provinces, respectively. Figure S2. UMAP visualization of wild and domesticated large yellow croaker based on selected InDel marker panels. (A) Large-fragment InDel panel. (B) Amino-acid-altering InDel panel. Both panels showed population-related grouping trends, but visual overlap remained between wild and domesticated samples.

## Abbreviations

AUC	Area under the ROC curve
DNA	Deoxyribonucleic acid
DNB	DNA nanosphere
Fst	Fixation index
InDel	Insertion/deletion
PCA	Principal component analysis
PCR	Polymerase chain reaction
RNA	Ribonucleic acid
ROC	Receiver operating characteristic
SNP	Single-nucleotide polymorphism
UMAP	Uniform Manifold Approximation and Projection
