# Supplementary material for: Development of InDel-Based Identification Methods for Wild and Domesticated Populations: A Case Study of Larimichthys crocea
Source: Animals (Basel). 2026 Jul 31;16(15):2339. doi: 10.3390/ani16152339 (PMC13463559; doi:10.3390/ani16152339)
Supplement: Supplementary file 1 [file animals-16-02339-s001.zip › animals-4366996-supplementary.pdf]

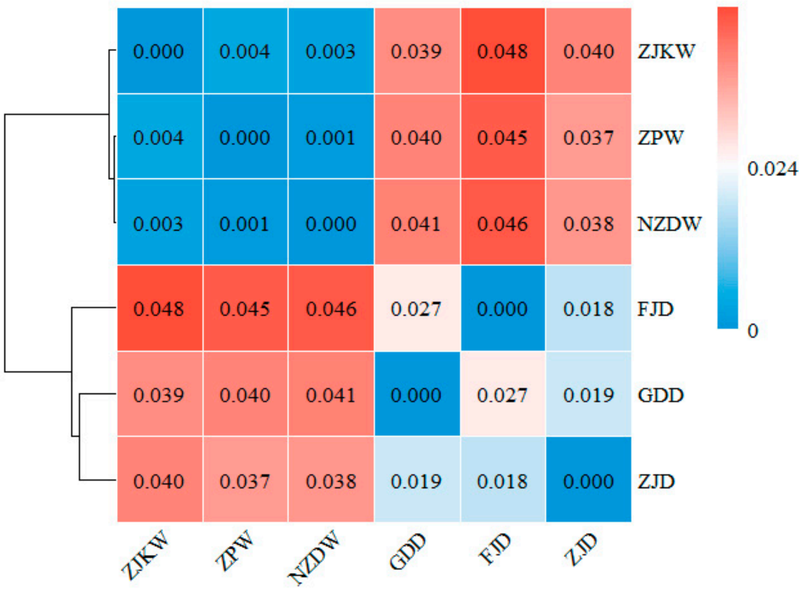

Figure S1. Genome-wide pairwise Fst among the six sampling populations of large yellow croaker used for marker discovery. Pairwise FST values were calculated using quality-controlled genome-wide SNPs. ZJKW, ZPW, and NZDW represent wild populations collected from the coastal waters near the Pearl River Estuary, the offshore areas of Liu’ao Town in Zhangpu City, and the waters near Naozhou Island, respectively. GDD, FJD, and ZJD represent domesticated populations from Guangdong, Fujian, and Zhejiang Provinces, respectively.

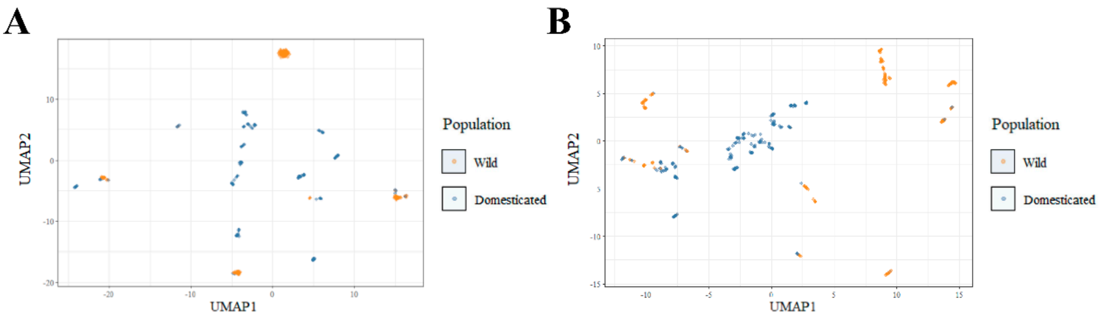

Figure S2. UMAP visualization of wild and domesticated large yellow croaker based on selected InDel marker panels. **(A)** Large-fragment InDel panel. **(B)** Amino-acid-altering InDel panel. Both panels showed population-related grouping trends, but visual overlap remained between wild and domesticated samples.

Table S1. SNP-based Fst and nucleotide diversity signals associated with the four selected amino-acid-altering InDels

InDel ID	InDel position	Gene	Supporting SNP-based window	Weighted Fst	Breed π	Wild π	πWild/πBreed	log2(πBreed/πWild)	Fst top 2%	π ratio top 5%	Joint Fst–π support	Signal summary
E1	11-5053756	IL-31RA	Chr11:5,010,001–5,060,000	0.123955	0.000646	0.000621	0.960714	0.057821	Yes	No	No	Fst-supported
E2	20-14004876	TP53RKB	Chr20:13,960,001–14,010,000	0.095820	0.000982	0.001089	1.108439	-0.148529	No	Yes	No	π-ratio-supported
E3	3-28345321	GIT2	Chr3:28,340,001–28,390,000	0.041083	0.001191	0.001261	1.059145	-0.082900	No	Yes	No	π-ratio-supported
E4	21-17327392	ctsh	Chr21:17,320,001–17,370,000	0.123744	0.000852	0.000920	1.079751	-0.110699	Yes	Yes	Yes	Fst- and π-ratio-supported

Table S2. Amplification primers used to verify the different InDels between wild and domesticated large yellow croaker based on allele frequency and chi-square test ($P < 0.001$)

InDel position (Chromosome-position)	Primer number	Primer sequence (5'-3')	In Amplification length (bp)	Del Amplification length (bp)	Fragment difference (bp)
21-17662246	D1-F	GTCTGAAGGAGAGACAGATCAGGA	316	263	53
	D1-R	CGGTGTATCTCATTGATATAATCTGA			
21-13979637	D2-F	GCAGCCTAGAGGTGGAAGAGAT	193	138	55
	D2-R	TCTTCTTTCTGTAAGTTTTAATGCG			
	D2-R*	TGAAGKTATGTGAAGTTTTAATGCG			
1-35741636	D3-F	AACCGAATCAGTAAAAAGACTCGT	385	332	53
	D3-R	GCTATTCTCGGGGTAATTGTGA			
3-21732774	D4-F	CACACGCTGCACATAGAGGC	263	213	50
	D4-R	GGGTGTCCCTAAAACTGGCAA			

Table S3. Amplification primers for differentiated InDels between wild and domesticated large yellow croaker based on verification and selective signal analysis

**InDel position (Chromosome-position)**	**Primer number**	**Primer sequence (5'-3')**	**Amplification length（bp)**	**Corresponding detection fragment type**
11-5053756	E1-F	GGTGCTCAATAAGACTCTGGTCAA		
	E1-dR4	ACTCCAGGGGGCCKCATTA	116	Del
	E1-iR4	ACTCCAGGGGGCCKCATCT	116	In
20-14004876	E2-F	TCTAAGTTAACAGCTTTGTTGGCA		
	E2-dR	TTTCCTGACCACAAAGTGACTCA	286	Del
	E2-dR2	TTTCCYGACCRCARAGTGACTCA	286	Del
	E2-iR	TTTCCTGACCACAAAGTGACCTC	286	In
	E2-iR2	TTTCCYGACCRCARAGTGACCTC	286	In
3-28345321	E3-F	GMTACACAGCAGAAACAATCACC		
	E3-dR2	CAGGCACAGTTTTATTTTCCTCAC	219	Del
	E3-iR2	CCAGGCACAGTTTTATTTTCCTATA	220	In
21-17327392	E4-F	AAAGTGAAAGTTACCGCGAACA		
	E4-dR	ATGTTTCACACTGATTGTTTGAGC	288	Del
	E4-iR	GTTATGTTTCACACTGATTGTTTGTTT	292	In

Note: Because there are deletion mutation sequences in the site primers, adding a sequence matching the deletion mutation sequence in the primer region in the reaction system is necessary. For example, if the E1 primer is used as an example, the E1-iR4

and E1-dR4 primer region deletion mutation sequence match, and they need to be added to the amplification system together with E1-dR4. In the table, Y is a degenerate base of C/T, K is a degenerate base of G/T, R is a degenerate base of G/A, and M is a degenerate base of A/C.
